# Annotations

**Published:** 1905-03-25

**Authors:** 


### Annotations.

### Alcohol and Commercial Efficiency.

The City of London has recently been entertained by an endeavour, on the part of a small number of advocates of total abstinence from alcohol, to show that its ordinary use is not conducive to "commercial efficiency," or in other words, we presume, to show that a teetotaller would be likely to make a better bargain in the market than a moderate drinker. Most people would be in agreement with the proposition that the custom of taking "drinks" after business transactions is a bad one, especially when the transactions of the drinker are small and numerous, and the drinks themselves are likely to be numerous in proportion. This custom, however, would in the first place remove those subject to it from the category of moderate into that of free drinkers, and, in the next place, would be less likely to prevail in the "City," where the transactions are large and are conducted by weighty and responsible persons, than in provincial fairs, markets, or public houses. It is well known that vast quantities of foreign and colonial produce are periodically sold in the City by auctions conducted at certain public marts, that the prices quoted are for small quantities, while the transactions are for very large ones, so that the difference of a fraction of a farthing in the pound will represent a very considerable difference in the total amount invested by the purchaser, and the biddings are extremely rapid. At all these sales there are well-known buyers of special expertness, sometimes of palate, sometimes of touch, according to the character of the goods dealt with; and there could be few better tests of "commercial efficiency" than many of these sales afford. It would be interesting to know in what proportion the best and most rapid judges are total abstainers; and the facts, which ought not to be difficult of discovery, would certainly show whether the moderate use of alcohol has any tendency to diminish the acuteness of sense perception. In connection with the general question involved, the final conclusion reached by the late Sir James Paget is one which ought not to be forgotten. He pointed out that, in this country, drinkers to excess and teetotallers had never been more than two small minorities, which might fairly be set off against each other. If this were done, it left the great majority of the British race the descendants of many generations of moderate drinkers; and, when they were so regarded, the facts of history showed that moderate drinking must be less of an evil than some people had supposed.

### Plague and Inoculation in India.

There is no doubt that the outbreak of plague in India this year is more serious than it was twelve months ago, and that the mortality, as disclosed by the official returns, is alarming. Dr. Klein urges that in consequence of the gravity of the situation, "the India Government should take drastic measures to insist upon inoculation with Dr. Haffkine's anti-plague serum." We do not think that Dr. Klein has sufficiently considered the difficulties which have to be encountered in enforcing this remedy. The prejudices of the natives in respect to inoculation have been most strongly aroused, and owing to the barriers of caste and the sanctity of the harem, it is next to impossible to carry it into effect. The Englishman rightly regards his house as his castle, and the Indian not only claims the same privilege, but guards it with keener jealousy, especially against the intrusion of the white man. It is well known that the attempts on various occasions to prosecute house-to house visitation in order to ascertain whether there were cases of plague, aroused vehement protests, and, in several instances, open resistance. The authorities therefore would seem to be face to face with the alternative of provoking local riots which might have disastrous and far-reaching consequences, or of allowing the disease to run its course. It may be hoped that the natives will be taught in process of time to recognise that any measures to check the progress of the disease are instituted in their own interest, but so long as they maintain their present attitude, and prefer the risk of death to the idea of inoculation, we are afraid that insistence is impracticable.

### The Association of Scottish Medical Diplomates.

This newly-founded association has for its general object the protection and advancement of the interests of those holding the medical and surgical diplomas of the Scottish Colleges. As is the case with the corresponding English Colleges, the Scottish licentiates and members have little or no voice in the governing councils of these institutions, and take no part in the election of the several representatives in the General Medical Council. It is urged that reform in both of these respects would be not only to the advantage of the diplomates themselves but also of the colleges and the profession generally. The new proposals are in short parallel to those which have for years occupied, perhaps almost exhausted, the time and energies of the association of the members of the English College of Surgeons, and may thus expect to receive the general sympathy of many English practitioners. In other directions the new Association may find its ambitions countenanced by not undistinguished authority. Thus it proposes to advance the right of every diplomate or licentiate to the use of the title "Dr." apart from the possession of the M.D. degree, and here it may find support in the scheme which the British Medical Association hopes to submit to Parliament for the reform of the General Medical Council and the profession generally. Another grievance which the Scottish Association desires to redress is the restriction, so common in England, of hospital appointments to the holders of English or rather of London qualifications—a policy in which also the Association may expect considerable sympathy and support. Altogether, and apart from minor details in the Association's programme, it cannot but be an advantage that those who own a common academic tradition should cultivate a professional association which, whilst specially directed to particular interests, must promote the general welfare by increasing the reasonableness and good understanding that arise from frank discussion and mutual conference.

